# GATA4 and GATA5 are essential for heart and liver development in *Xenopus* embryos

**DOI:** 10.1186/1471-213X-8-74

**Published:** 2008-07-28

**Authors:** Kim E Haworth, Surendra Kotecha, Timothy J Mohun, Branko V Latinkic

**Affiliations:** 1 School of Biosciences, Cardiff University, Museum Avenue, Cardiff, CF10 3US, Wales, UK; 2 Division of Developmental Biology, National Institute for Medical Research, The Ridgeway, London NW7 1AA, UK

## Abstract

**Background:**

GATA factors 4/5/6 have been implicated in the development of the heart and endodermal derivatives in vertebrates. Work in zebrafish has indicated that GATA5 is required for normal development earlier than GATA4/6. However, the GATA5 knockout mouse has no apparent embryonic phenotype, thereby questioning the importance of the gene for vertebrate development.

**Results:**

In this study we show that in *Xenopus *embryos GATA5 is essential for early development of heart and liver precursors. In addition, we have found that in *Xenopus *embryos GATA4 is important for development of heart and liver primordia following their specification, and that in this role it might interact with GATA6.

**Conclusion:**

Our results suggest that GATA5 acts earlier than GATA4 to regulate development of heart and liver precursors, and indicate that one early direct target of GATA5 is homeobox gene Hex.

## Background

GATA4/5/6 Zinc finger-containing transcription factors are regulators of gene expression in multiple tissues derived from mesoderm and endoderm in vertebrate embryos and adults [1-4]. They are expressed in overlapping patterns and share domain structure and most of their biochemical activities, including DNA binding site preference, raising a possibility of redundant function in vivo. Genetic analyses of the roles of GATA4/5/6 genes in vertebrate development has so far implicated that individual factors have unique and shared roles. In the mouse GATA4 initially plays an essential role in endodermal development and indirectly in early heart morphogenesis [5-7]. Later roles of GATA4 have been revealed by analysis of a non-null allele that blocks interaction with FOG2 cofactor, by myocardial-specific null mutation, and by careful analysis of heterozygous null mice [8-10]. A role for GATA4 in the mouse liver development was recently examined by tetraploid embryo complementation, and this work has indicated that GATA4 is required for expansion of liver bud [11]. In zebrafish, knockdown of GATA4 by specific morpholino oligonucleotides (MO) has been shown to affect development of heart and liver precursors after their specification, in broad agreement with the results obtained in the mouse model [12].

GATA6 is indispensable for development of exrtraembryonic endoderm in the mouse, and also has later roles in the developing liver and heart, following their specification [13]. These findings on the roles of GATA6 in the mouse are in good agreement with the results from *Xenopus *and zebrafish models [12,14].

The function of GATA5 has been most extensively studied in the zebrafish embryo, where *faust *(GATA5) mutants were found to display defects in heart and endoderm development [15,16]. The heart phenotypes of *faust *embryos include an almost complete loss of cardiac progenitors and morphological defects such as cardia bifida [15]. More recently a role of GATA5 in zebrafish heart morphogenesis was confirmed by MOs [17]. In contrast, targeting of the GATA5 gene in the mouse was reported to have no consequences for embryonic development, suggesting that the role of GATA5 in mammals may not be conserved [18].

To improve our understanding of the roles played by GATA4 and GATA5 in early development of vertebrate embryos, we have analysed their function in *Xenopus *embryos using a gene knockdown approach.

## Results

### GATA5 is a dosage-sensitive regulator of early heart and liver development

**Figure 1:**
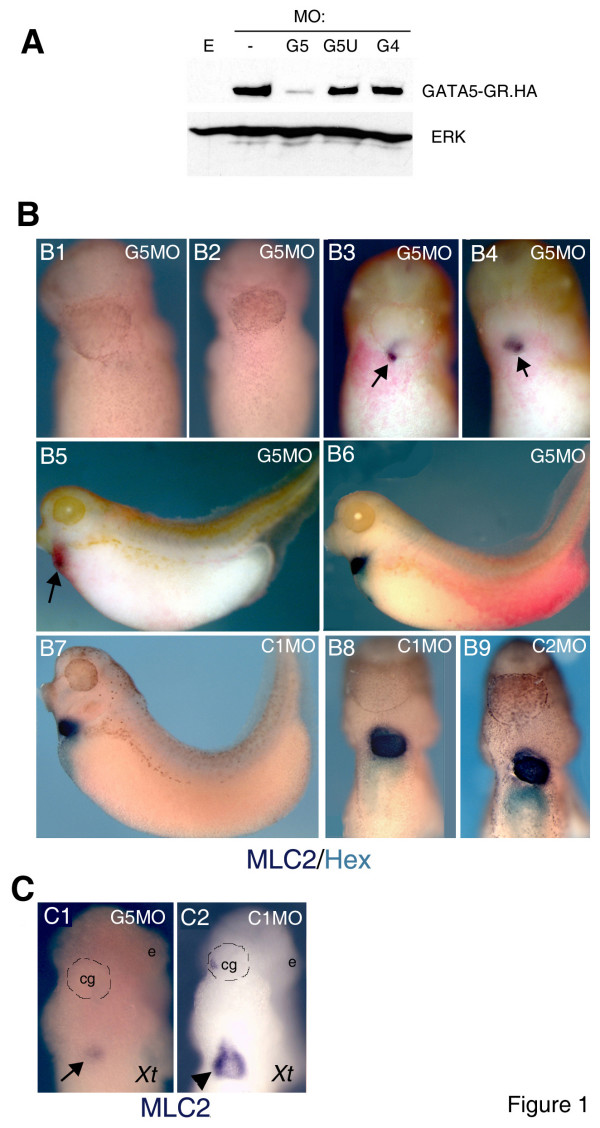
**Injection of G5 MO, but not of C1 and C2 control MOs, cause severe size reduction or loss of heart and liver in Xenopus embryos**. **A: **Translation of injected GATA5-GR.HA mRNA, detected by Western blotting with anti-HA antibody, is blocked by G5 but not by other MOs indicated. G5UTR MO ("G5U") does not affect translation of GATA5-GR.HA, as this constructs lacks the 5'UTR sequence. 1 ng of mRNA was injected into 1- or 2-cell embryos and 10–15 minutes later the embryos were injected with 10 ng of G5, or 50 ng of G5UTR or G4 MOs. E-Uninjected embryos. **B: **Injection of 5 ng of G5 MO results in a loss (B1,2) or severe reduction (B3-5) of cardiac and liver precursors, as revealed by whole-mount in situ hybridisation for MLC2 (purple) and Hex (turquoise). Posterior injection of G5 MO (5ng) has no obvious effects (B6). Control MOs 1 or 2 (C1 or C2, 50 ng/embryo) have no effect on normal development of heart and liver precursors (B7-9). B1-4, 8,9: ventral view; B5-7: lateral view (anterior to the left). In B3-5 arrows point to the remnants of the heart. **C: **G5 MO (1 ng/embryo) causes heart defects in *X. tropicalis *(*Xt*) embryos (arrow points to the heart remnant), and C1 MO (10 ng) has no effect on normal heart development (arrowhead in C2). Complete bleaching of *X. tropicalis *embryos has removed the morphological landmarks, and cement gland (cg) and eyes (e) have been indicated to add visualisation.

To assess the role of GATA5 in *Xenopus *development we first used a translation-blocking MO, G5. This MO targets both GATA5 pseudoalleles in *Xenopus laevis *and GATA5 mRNA in diploid *Xenopus tropicalis *(see Additional File 1). Doses of 20 ng or greater per *X. laevis *embryo resulted in gastrulation defects and lethality by mid-neurula stages. Therefore, to investigate the role of GATA5 in heart and liver development doses of 5–10 ng/embryo were used. This dose of G5 MO was shown to specifically and effectively block translation of the injected tagged GATA5 mRNA (Fig. 1A).

The phenotypes of morphant tadpoles were scored at st. 35–37, when morphogenesis of the beating heart is well advanced, and is characterised by the expansion of the walls of looped cardiac tube [19]. At this stage the adjacent liver primordium is visible as a distinct thickening in the anterior endoderm. At st. 35–37, the cardiomyocyte differentiation-specific genes MLC2 and cTnI, and liver-enriched genes FOR1 and Hex are robustly expressed, and were used to visualise heart and liver development [20-23].

Injection of G5 MO in *X. laevis *embryos in both blastomeres at 2-cell stage or in presumptive anterior blastomeres at 4-cell stage caused a severe reduction in the size of heart and liver primordia (Fig 1B1-5), whilst posterior targeting of G5MO (Fig 1B6) or injection of two control MOs at 50 ng/embryo has no obvious effect (Fig. 1B7-9). G5 MO caused a similar heart phenotype in *X. tropicalis *embryos (Fig 1C).

The most frequent phenotype caused by G5MO was an almost complete loss of cardiac and liver markers (101/146; summarised below). The remaining morphants had a reduction of heart and liver primordia, and only rarely abnormal morphogenesis of the heart was observed.

**Figure 2:**
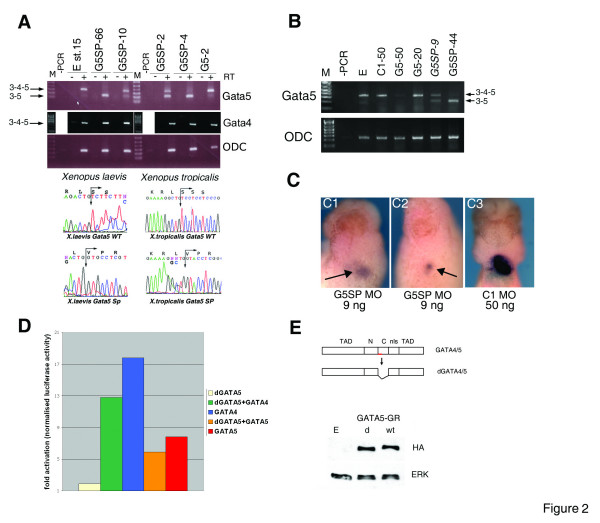
**G5SP MO creates GATA5 protein lacking the C-terminal Zn finger and causes heart and liver defects**. **A: **G5SP MO causes dose-dependent splicing out of exon 4 in both *Xenopus laevis *and *Xenopus tropicalis*. The injected dose is indicated (in ng). 3–4–5, cDNA that contains exon 4 and regions of exons 3 and 5 determined by target sites of the primers; 3–5, cDNA without exon 4. Below are shown the sequences of the wt 3–4–5 and 3–5 cDNAs showing in-frame splicing in both species. **B: **G5SP MO causes a dose-dependent reduction in the level of the wt full-length mRNA (including exon 4; 3–4–5) and concomitant increase in the level of the mRNA that lacks exon 4 (3–5), as revealed by RT-PCR with primers based in exons 2 and 4. Injection of 9 ng of G5SP MO causes partial loss (~50%) of wt GATA5 mRNA. The dose in ng used per embryo is given for each MO. -PCR, control with no cDNA input. M-DNA marker. ODC- Orhithine Decarboxylase loading control. Embryos were collected for RNA analysis at st. 15. **C: **Injection of 9 ng of G5SP MO into the same group of embryos analysed in (B) causes severe reduction of heart and liver st. 37 (C1,2). Injection of 50 ng of C1 MO has no effect on heart and liver development (C3). **D**: The dGATA5 protein can neither activate transcription nor can it significantly affect the ability of GATA5 or GATA4 to activate a firefly luciferase reporter driven by 2 GATA sites in animal cap explants. Dual luciferase assays were performed 3 hours after excision of explants, and firefly luciferase activity was normalised to renilla luciferase activity resulting from TK-RL DNA. A representative experiment (out of 3) is shown; whilst the levels of induction varied between experiments, the trend (activation by GATA4 or GATA5 and lack of substantial effect by dGATA5) remained consistent. **E: **Schematic representation of the effect of G4/5SP MOs (exon-specific part shown as red line) on the domain structure of their targets. TAD-Trans Activation Domain; NLS-Nuclear Localisation Signal; N, C-Zn fingers. Below-Western blot showing efficient translation of the dGATA5 protein in embryos, detected with anti-HA antibody. E-uninjected embryos. d, wt-embryos injected with the d- or wtGATA5-GR.HA mRNA, respectively.

Our attempts to rescue GATA5 morphants by injection of a wide range of GATA5 mRNA amounts proved inconclusive (data not shown), most likely because overexpression of even small amounts of GATA5 mRNA causes a severe phenotype [24]; data not shown). It is likely that a successful rescue requires the precise regulation of the amount, place and time of exogenous GATA5 expression. We therefore wished to provide an additional line of evidence for the specificity of action of GATA5 MOs by downregulating GATA5 in a way that would enable assessment of the status of endogenous GATA5. To achieve this, a splice site-blocking MO was used and its action was monitored by RT-PCR analyses of mRNA. Using genomic sequence available for *X. tropicalis*, a splice junction blocking MO (G5SP) was designed which was predicted to cause in-frame skipping of exon 4. This exon encodes the C-terminal Zn finger (Fig. 2). Injection of G5SP MO caused an efficient and dose-dependent reduction in the level of the full-length GATA5 mRNA in *X. tropicalis *embryos (Fig. 2A). This MO also caused exon skipping in *X. laevis *(Fig. 2A, B), indicating conservation of intronic sequence at the exon/intron and intron/exon boundaries (currently *X. laevis *GATA5 genomic sequence is not available to confirm this assumption).

Injection of G5SP MO in both *X. laevis *and *X. tropicalis *resulted in correct in-frame exon 4 skipping, as confirmed by sequencing of PCR products (Fig. 2A). The action of G5SP MO appeared to be specific for GATA5, since GATA4 transcripts were not affected by any of the doses of this MO analysed (Fig. 2A). Importantly, the phenotype observed following injection of the low-medium doses (9–18 ng/*X. laevis *embryo) was similar to the one produced by G5 MO, namely, severe heart and liver defects (Fig. 2C). Furthermore, given that 9 ng of G5SP MO eliminated only approximately half of the full-length GATA5 mRNA (Fig. 2B), these results suggest that in our experiments with translation-blocking MO, only a partial downregulation of GATA5 was achieved, allowing the morphants to reach tadpole stages. RT-PCR data suggest that doses of G5SP MO above 18 ng (44 and 66 ng) result in skipping of the exon 4 in all GATA5 transcripts in *X. laevis *(Fig. 2A, B) and cause severe gastrulation defects and death (data not shown). In our earlier experiments with higher doses of the G5 MO (20–50 ng) we observed similar phenotypes. Taken together, this data indicate that higher doses of G5SP and G5 MOs result in complete or nearly complete loss of GATA5 and lethality.

The action of G5SP MO results in the formation of mutant GATA5 protein with a deleted C-terminal Zn finger, dGATA5 (Fig. 2E). Given that the C-terminus Zn finger is sufficient for DNA binding of GATA5 [25], we expected that the dGATA5 protein would be inactive. This was confirmed by showing that dGATA5 is unable to activate GATA transcriptional reporter in animal cap explants (Fig. 2D). The dGATA5 protein was efficiently expressed in animal caps, indicating that its lack of transcriptional activity is likely due to loss of DNA-binding Zn finger, rather then altered protein levels (Fig 2E). dGATA5 retains most of the wt coding sequence, including the activation domains and the N-terminal Zn finger. In order to test if dGATA5 retained any activity which enabled it to interact with factors such as the wild-type GATA5 or GATA4, we modelled the potential interaction between the wtGATA4/5 and dGATA5 proteins, as it may occur in vivo in G5SP morphants where the MO was 50% effective in generating the dGATA5 protein. An equal amount of mRNAs encoding wtGATA4/5 and dGATA5 proteins was co-injected into embryos together with a GATA transcriptional reporter. We found that dGATA5 does not substantially alter transcriptional activity of either GATA5 or GATA4 in animal caps (Fig. 2D). These results suggest that the phenotype caused by the low doses of G5SP MO (9–18 ng) likely results from a reduction in the amount of the wt protein.

**Figure 3:**
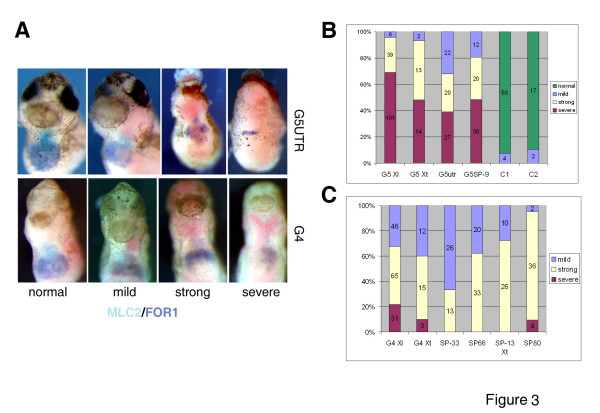
**Summary of effects of GATA4 and GATA5 MOs in Xenopus embryos**. **A: **Examples of phenotypic classes caused by GATA5 and GATA4 MOs. Ventral views of embryos injected with G5UTR MO or G4 MO are shown (50 ng/embryo). Heart and liver precursors have been revealed by MLC2 (BCIP) and FOR1 (BM purple) probes, respectively. FOR1 was developed first. Ventral views are shown, with anterior at the top. **B, C: **Summary of frequencies with which the heart and liver phenotypes were observed for GATA5 MOs (B) and for GATA4 MOs (C). The doses of splicing MOs are indicated and for other MOs are as in Figs. 1 and 4.

An additional non-overlapping translation-blocking MO, G5UTR also caused deficiency in heart and liver tissue (Fig. 3). However, G5UTR MO was less effective (27/69 severely affected embryos; Fig. 3B), requiring a 10 times greater dose then G5 MO to produce a similar phenotype. The difference in efficiency between these MOs might reflect the different location of their target sequences along the GATA5 mRNA: the G5 MO target is immediately downstream of the START codon, whereas the G5UTR MO interacts with its target 8 nt upstream (Additional Fig. 1), and its' efficiency might be affected by the 5'UTR secondary structure.

### GATA4 is required for heart and liver development in *Xenopus* embryos

**Figure 4:**
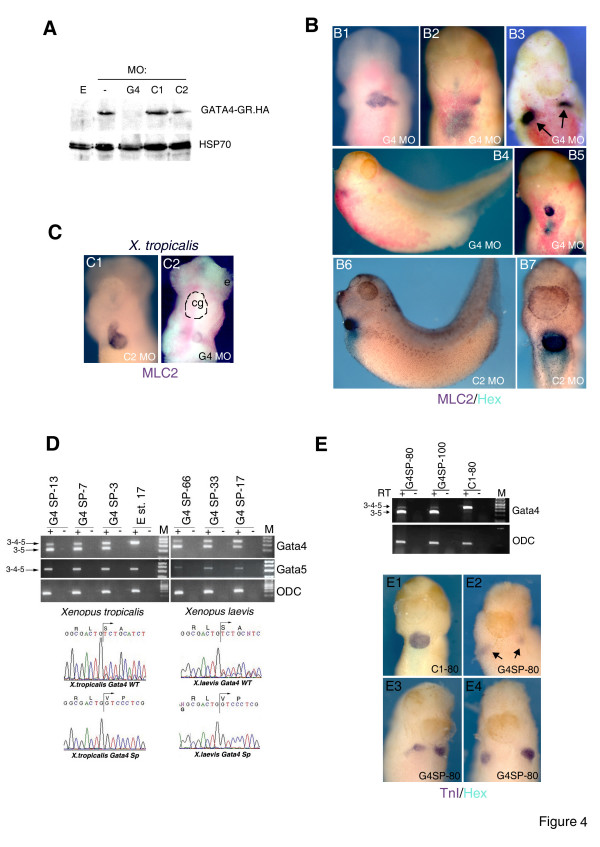
**GATA4 MOs cause defects in heart and liver development**. **A: **Translation of injected GATA4-GR.HA mRNA, detected by Western blotting with anti-HA antibody, is blocked by G4 but not by other MOs indicated. 1 ng of mRNA was injected into 1- or 2-cell embryos and 10–15 minutes later the embryos were injected with 50 ng of indicated MOs. E-Uninjected embryos. **B: **Injection of 50 ng/embryo of G4 MO leads to a reduction in heart and liver precursors and to cardiac morphogenesis defects such as cardia bifida, highlighted by arrows in B3 (B1-5). The same dose (50 ng/embryo) of the C2 MO has no effect on heart and liver development (B6,7). B1-3,5,7: ventral view. B4,6: lateral view. Heart and liver precursors were revealed by MLC2 and Hex probes (BM purple and BCIP, respectively). **C: **10 ng/embryo of C2 MO has no effect on heart in *X. tropicalis *embryos, but the same dose of G4 MO causes heart defects. **D: **G4SP MO causes dose-dependent splicing out of exon 4 in both *Xenopus laevis *and *Xenopus tropicalis*. The injected dose is indicated (in ng). 3–4–5, cDNA that contains exon 4 and regions of exons 3 and 5 determined by target sites of the primers; 3–5, cDNA without exon 4. Below are shown the sequences of the wt 3–4–5 and 3–5 cDNAs showing in-frame splicing in both species. **E (top): **Injection of 80 or 100 ng/embryo of G4SP MO, but not of 80 ng of C1 MO, causes splicing out of exon 4 with ~90% efficiency, as detected by RT-PCR analyses of mRNA from st. 15 embryos. +/- RT-indicates presence or absence of Reverse Transcriptase in samples that were analysed by PCR.**E (bottom): **80 ng of C1 MO has no effect on heart and liver development (E1), whereas the same amount of the G4SP MO causes cardia bifida and liver defects (E2-4). In E2 remnants of cardiac tissue detected by weak expression of cardiac marker in severely affected embryo with cardia bifida are shown by arrows. Ventral views are shown. Heart was labelled by cTnI (purple) and liver with Hex (light blue/turquoise) probes.

GATA4 was shown in both zebrafish [12] and mouse [11,26,27] to be essential for heart and liver development following specification, but its role in *Xenopus *has not been investigated in detail. To address this question we designed a MO targeted against both *X. laevis *alleles and *X tropicalis *GATA4 (Additional File 1). This MO, G4, causes specific blocking of translation of injected mRNA (Fig. 4A) and results in heart and liver defects (Fig. 4B and also Fig. 3). The majority of G4 morphants had heart morphogenesis defects but had detectable heart and liver gene expression (Figs 3, 4). However, in these experiments the status of endogenous GATA4 in G4 morphants was unknown. Therefore, we additionally tested the role of GATA4 by using a splice-blocking MO, G4SP, which was designed like the G5SP MO to cause in-frame removal of exon 4 in its target. As shown in Fig. 4D, G4SP MO caused a dose-dependent splicing out of exon 4 in both *X. tropicalis *and *X. laevis*, resulting in heart and liver defects. G4SP morphants closely resembled G4 morphants (Fig. 4). However, the maximum activity of the G4SP MO (~90% efficiency in inducing splicing) was reached at 80 ng/embryo (Fig. 4D, E), unlike G5SP MO, which was effective in causing complete splicing above 18 ng/embryo. Further increase in the dose of the G4SP MO to 100 and 120 ng/embryo did not improve the efficiency of splicing (Fig. 4E and data not shown). This result was observed in both *X. laevis *and *X. tropicalis *(Fig. 4C) and therefore likely represents inherent property of the G4SP MO, rather then inefficient action caused by sequence divergence. G4SP MO did not cause splicing out of exon 4 in GATA5 mRNA (Fig. 4D).

Importantly, the phenotype caused by the doses of G4SP MO above 33 ng closely resembled the phenotype of G4 morphants (Fig. 4), suggesting that loss of greater than 50% of wt GATA4 leads to heart and liver defects. The effect of G4SP MO was dose dependent, with the proportion of embryos with cardiac and liver defects increasing from 33% at 33 ng/embryo to 95% at 80 ng/embryo (Fig. 3). The GATA4 MOs were far less effective in causing severe phenotype (severe reduction or loss of heart and liver precursors) than the MOs against GATA5 (summarised in Fig. 3). The great majority (80–90%; Fig. 3) of GATA4 morphants displayed defects in expansion or morphogenesis of the heart primordium, including cardia bifida, partial fusion of heart fields and abnormal looping. These results suggest that GATA4 is required for heart development after cardiac specification.

### GATA5 is required before GATA4 for development of heart and liver precursors

**Figure 5:**
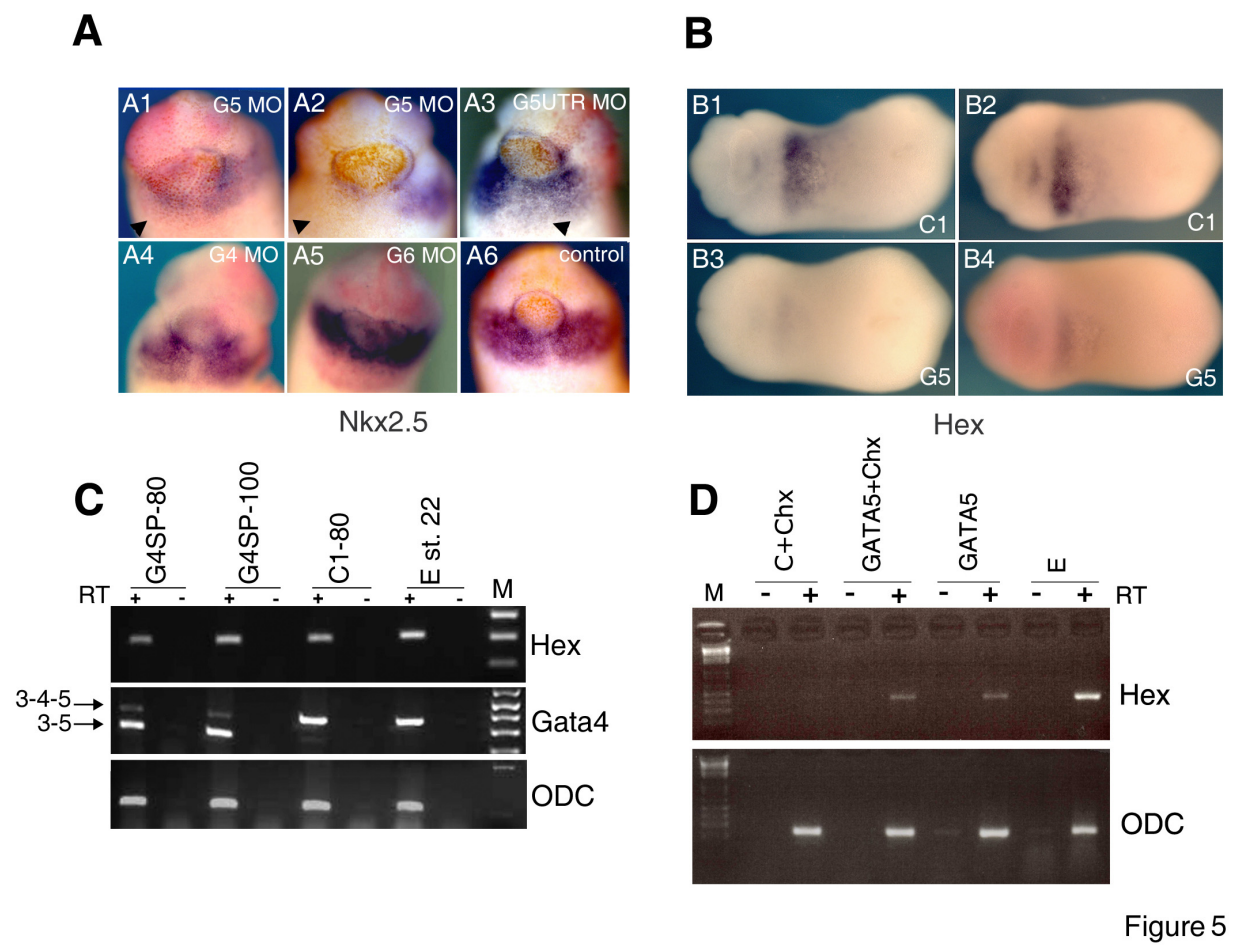
**GATA5 is required for stable specification of heart and liver precursors and is sufficient to directly induce Hex**. **A: **GATA5 MOs (20/32 embryos for G5 (5 ng) and 14/25 embryos for G5UTR (50 ng) showed reduced expression), but not GATA4 MOs (19/19 embryos for G4 (50 ng) and 22/22 embryos for G4SP (100 ng) expressed Nkx2.5) or GATA6 MO (10 ng; 14/14 embryos with normal expression), cause early deficiency of heart precursors at st. 22 (arrowheads in A1-3). Heart field was revealed by Nkx2.5 expression. Ventral views are shown, with anterior at the top. **B: **Liver precursors in anterior endoderm (see Additional File 3) expressing Hex are affected by 10 ng of G5 MO (31/39), but not by 50 ng of C1 MO (20/23 normal). Embryos injected uniformly are shown before (B1,3) and after (B) staining for lineage tracer (pink). Ventral views are shown, with anterior to the left. **C: **G4SP MO does not effect Hex expression. Embryos injected with indicated MOs were analysed at st. 22 for expression of Hex, splicing out of exon 4 of GATA4 and for ODC by RT-PCR. **D: **GATA5-GR induces expression of Hex mRNA in animal caps explants in the presence or absence of cycloheximide (Chx), which was added at st. 9. All samples were treated with dexamethasone at the same time to activate GATA5-GR protein. E-st. 11 control embryos.

To determine when GATA4 and GATA5 are required for heart development, we have examined the expression of Nkx2.5, a marker of heart field in late neurula stages, when it is robustly expressed [28,29]. Another reason for performing the analysis at st. 22 was that previous fate mapping studies have shown that at st. 22 Nkx2.5-expressing cells contribute to the developing heart [29]. We found that injection of any GATA5 MO, but not GATA4 or GATA6 MOs [14], cause a substantial reduction in expression of Nkx2.5 (Fig. 5A).

The expression of another marker which is expressed in a subset of cardiac precursors at the same time, Tbx5 [30], was also reduced by GATA5 MO (Additional file 2), providing further evidence that GATA5 is required for specification of cardiac precursors. We have additionally tested the effect of GATA5 MOs on Nkx2.5 expression at st. 14, near its' onset, [28] and just after cardiac specification [31]. Our RT-PCR analyses indicate that GATA5 MOs do not block expression of Nkx2.5 at st. 14 (data not shown). However, as early-expressing Nkx2.5-positive cells have not been fate mapped, and, more importantly, since there are no known cardiac-specific markers that uniquely identify cardiac precursors at the time of their specification, the earliest requirement for GATA5 in heart development remains unknown at present.

To examine the effect of GATA4 and GATA5 MOs on early liver precursors, we first established that Hex-expressing cells in the anterior ventral endoderm of st. 22 embryos are fated to become the liver primordium (Additional File 3). We next established that injection of G5 MO (Fig. 5B), but not of control MO or GSP4 MO (Fig. 5B, C), leads to an almost complete loss of Hex expression. This result suggests that GATA5 is required for liver specification. However, additional specific markers are required to establish the requirement of early liver precursors for GATA5.

The same dose of G5 MO that causes a reduction of Hex expression at st. 22 and later cause heart and liver defects in tadpoles had no effect on Hex expression in gastrulae (data not shown). However, higher doses of G5 and G5SP MOs (that are more effective in downregulation of GATA5; see above) lead to a reduction of Hex expression at st 11 (Additional file 4). These results indicate that GATA5 regulates Hex in time- and dose-dependent manner.

We have additionally established that GATA5 is likely to be a direct regulator of Hex expression, as it is sufficient to induce Hex mRNA in an immediate-early fashion in gastrula-stage animal caps (Fig. 5D). At this stage Hex expression marks anterior endoderm [23]. Whether Hex is also an immediate-early target of GATA5 in liver precursors at st. 22 is not known.

GATA5 is expressed in endoderm during gastrulation and subsequently during neurula and early tailbud stages in mesoderm and anterior endoderm [24]. In our experiments G5SP MO has been shown to be effective until tadpole stages (Additional File 5), having the potential to affect both early and late phases of GATA5 expression, which could both be involved in normal development of heart and liver. Therefore, at present it is not clear when precisely GATA5 acts in the development of heart and liver precursors.

### Endodermal GATA4 and GATA5 are required for normal heart development

**Figure 6:**
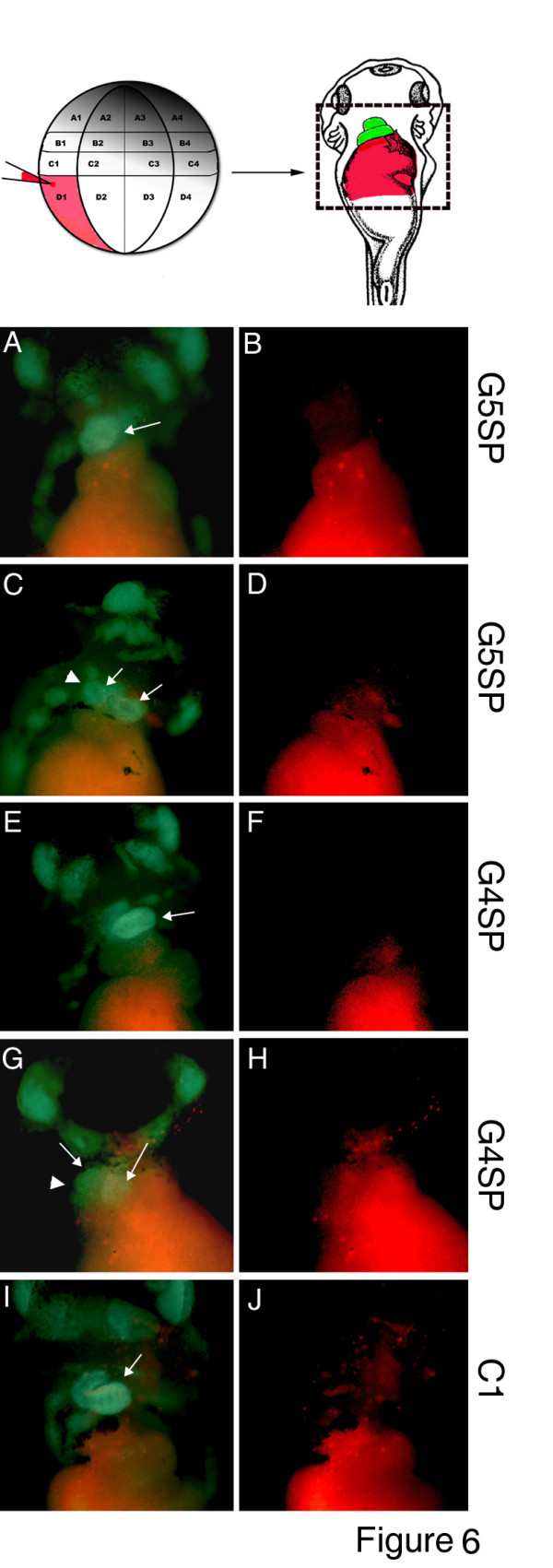
**Injection of GATA4 and GATA5 MOs in endoderm interferes with heart morphogenesis**. **Top: **experimental scheme showing targeted co-injections of MOs and rhodamine-dextran (red fluorescence). The drawing of ventral view of Nieuwkoop and Faber stage 41 embryo [55] is from . Cardiac actin/GFP embryos were injected at 32-cell stage with indicated MOs and the fate of injected cells was recorded at st. 41 by rhodamine fluorescence **(B, D, F, H, J)**. **A, C, E, G, I: **GFP fluorescence was used to observe heart development (cardiac actin/GFP is expressed in all striated muscles, and head muscles are visible in all images), and GFP images were merged with rhodamine images to highlight injected areas. Heart is indicated by arrows, and in specimens where heart was partially injected **(C, G)**, uninjected area is highlighted by an arrowhead. All properly targeted embryos were similarly affected by G4SP and G5SP MOs (n = 8, 10, respectively), but not by C1 MO (n = 7). Ventral views are shown, with anterior at the top.

GATA4 and GATA5 are expressed in both cardiac mesoderm and hepatogenic endoderm, and are required for their normal development. In our experiments we have downregulated GATA4 and GATA5 in both heart and liver precursors, and therefore the cell autonomy of their function was not addressed. To begin to address the specific roles of GATA4 and GATA5 in heart and liver precursors in more detail, we have performed mosaic analysis by targeting GATA4 or GATA5 MOs at 32/64 cell-stage to anterior-vegetal blastomeres, fated to contribute to anterior endoderm [32]. MOs were co-injected with rhodamine-dextran, and the fate of injected cells was followed from tailbud stages. This allowed us to examine heart development in real time in embryos with correct (largely anterior endoderm-specific) targeting. In those embryos heart development was examined by using cardiac actin GFP reporter transgenic line [33]. As shown in Fig. 6, the presence of both G4SP and G5SP MOs in anterior endoderm causes abnormal cardiac morphogenesis (defective looping (Fig. 6C, G) or poor expansion of the ventricle (Fig. 6A, E), suggesting that both factors can act in endoderm to affect the development of neighbouring heart primordium. In these experiments we have not observed more severe phenotypes that have been seen with uniform injection of the MOs (Figs 1, 2, 3, 4). However, given that it is difficult to specifically target all anterior endodermal precursors by injections, and since the efficacy of mosaically expressed MOs is unknown, our knowledge of the roles of endodermal GATA4 and GATA5 is still incomplete.

### GATA4 and GATA6 interact in heart and liver development in *Xenopus* embryos

**Figure 7:**
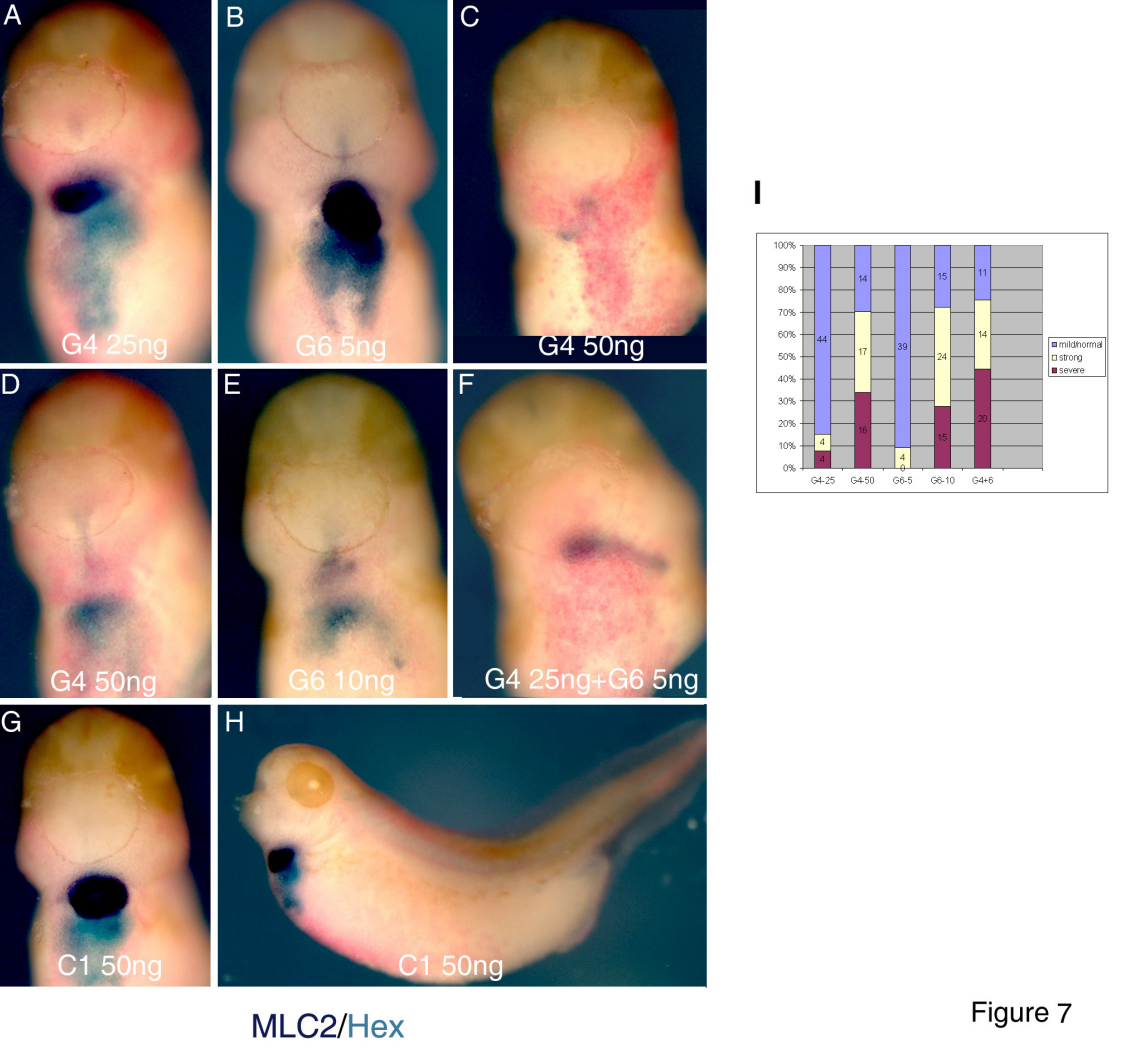
**Interaction between GATA4 and GATA6 in heart and liver development**. Suboptimal doses of G4 (**A**, 25 ng) or G6 (**B**, 5 ng) MOs have only minor effects on heart and liver development at st. 37. 50 ng of G4 MO (**C, D**) or 10 ng of G6 MO cause heart and liver defects. Similar defects are seen in embryos coinjected with 25 ng of G4 MO and 5 ng of G6 MOs (**F**). **G, H: **50 ng/embryo of C1 MO has no effect on heart and liver. **I**-frequencies of the phenotypes observed. Heart and liver precursor have been analysed as in Fig. 1 with MLC2 and Hex markers.

GATA4/5/6 are expressed in overlapping patterns in heart and endoderm in vertebrate embryos, and share many biochemical properties, including the domain structure and DNA binding site preference [1,3,4]. We have addressed the possibility of redundancy between GATA4 and GATA6, as their knockdowns cause a later and milder heart and liver precursor phenotype than a knockdown of GATA5 (Fig. 1, 2, 3, 4, 5; [14]. Whilst suboptimal doses of GATA4 and GATA6 (25 and 5 ng, respectively) have no effect or cause only a mild phenotype, their co-injection causes heart and liver defects that resemble the phenotype caused by 50 ng of the GATA4 MO or 10 ng of the GATA6 MO (Fig. 7). These results strongly suggest that GATA4 and GATA6 interact during development of heart and liver.

## Discussion

### GATA5 is essential for early heart and liver development in *Xenopus* embryos

In this report we have established that in *Xenopus *embryos GATA5 is required for the early development of heart and liver. Downregulation of GATA5 in both *Xenopus laevis *and *Xenopus tropicalis *using two non-overlapping translation-blocking MOs and by using a splice-site blocking MO causes a severe reduction in the number of heart and liver precursors at the time of or shortly after their specification. Our results are in broad agreement with the analysis of the *faust *(GATA5) zebrafish mutants, which show abnormal development of the heart and endodermal tissues, including the liver [15,16]. However, the genetic analyses of GATA5 in zebrafish have been limited by the fact that of two described *faust *alleles the stronger one remains uncharacterised at the molecular level, by considerable variability of the phenotype and by a possibility of maternal contribution [15].

A recent report has examined the roles of GATA4/5/6 in heart development of *Xenopus laevis *and zebrafish embryos [34]. In contrast to our findings, Peterkin et al. have concluded that in *Xenopus *embryos GATA5 only plays a relatively minor role in heart development, after specification. This discrepancy likely reflects different approaches taken to address the role of GATA5: whilst Peterkin et al. have used a single MO (which is identical to the least effective GATA5 MO (G5UTR) that we have used in 20 out of 25 nucleotides), we have used two non-overlapping translation blocking MOs in addition to a splice site-blocking MO to induce similar phenotypes in two species, *X. laevis *and *X. tropicalis*. In addition, we have also documented the efficiency of splice-site blocking MO by gene expression analyses. The data of Peterkin et al. on the role of GATA5 in zebrafish based on the use of both translation-blocking and splice-site blocking MOs have confirmed and extended the results obtained in *faust *mutants, that GATA5 is the most important and earliest-acting GATA factor in heart development and are in better agreement with our study. One difference, however, is that we found that high doses of G5 and G5SP MOs cause gastrulation defects and early lethality in *Xenopus*, whereas in the zebrafish GATA5 morphants have a milder phenotype [34]. At present it is not clear if this reflects a greater reliance of the frog embryo on GATA5 for early development, as it is also possible that the apparent difference reflects maternal contribution to GATA5 function in zebrafish. Clearly, a direct comparison and more complete answer about the role of GATA5 in *Xenopus *and zebrafish will require the generation and characterisation of embryos without GATA5 activity in both models.

Our results appear to disagree with the reported lack of embryonic phenotype of the GATA5 gene-targeted mice [18]. However, it has been suggested that in the mouse the targeting strategy might not have resulted in a null mutation [35], and it therefore remains to be established whether GATA5 plays a conserved role in early development of mammals.

The findings of our study strongly suggest that GATA5 is required very early in the development of cardiac and liver precursors, and are consistent with the reported ability of GATA5 to induce cardiac and endodermal tissue in *Xenopus *and zebrafish embryos [15,16,24,36]. Having found that GATA5 is essential for heart and liver development in *Xenopus *embryos, it will now be important to understand how it acts. Our initial experiments designed to address the cellular basis of GATA5 action in heart and liver development have suggested that GATA5 MOs do not significantly alter the pattern of apoptosis or proliferation in heart and liver precursors at the time when their numbers are greatly reduced (i.e., st.22; KEH and BVL, unpublished). Whilst it is possible that GATA5 MOs affect cell proliferation and apoptosis in heart- and liver-forming area of the embryo at an earlier stage, ultimately better knowledge of the mechanism of action of GATA5 will require identification of the target genes that mediate its' action. In this report we have begun this process by showing that homeobox gene Hex is an immediate-early target of GATA5, and we are currently extending this finding by conducting a systematic search for targets of GATA5.

### Dose-dependent roles of GATA5 in early development

In this report we have focused on the role of GATA5 in heart and liver development, however during the course of our work we have observed that GATA5 also plays an essential earlier role, during gastrulation. This early role was revealed using higher doses of G5SP MO which efficiently remove wt GATA5 mRNA (Fig. 2) and result in severe gastrulation defects and death (data not shown). However, one limitation of this result is that it remains to be formally established whether the action of the G5SP MO represents the equivalent of a null mutation, despite the fact that we found no evidence for activity of the mutant dGATA5 protein (Fig 2). A very similar early phenotype that likely results from a complete or nearly complete loss of GATA5 activity was obtained by higher doses of G5 MO (20–40 ng/embryo; Additional file 4 and data not shown), providing additional evidence that GATA5 has an essential role during gastrulation.

The two roles of GATA5 have a differential requirement for the level of GATA5 protein, with the development of heart and liver precursors being more sensitive. A part of our effort to identify targets of GATA5 is currently focused on isolation of subsets of targets that require different levels of GATA5 activity. An interesting precedent for dose-dependent action of GATA factors has recently been established for GATA1, which has been shown to directly regulate subsets of target genes that differentially respond to its levels [37].

### The role of GATA4 in heart and liver development in *Xenopus* embryos

Our work shows that in *Xenopus laevis *and *Xenopus tropicalis *GATA4 is required for development of heart and liver precursors after their specification. This has been established by using a specific translation-blocking MO and a splice-site blocking MO that operates in a similar manner to G5SP MO, resulting in the removal of exon 4. However, unlike G5SP MO, which at doses that cause approximately 50% splicing of its target induces severe heart and liver defects, the maximal activity of the G4SP MO, 90% of splicing of its target, results in a milder phenotype, characterised by heart morphogenesis defects. As mentioned above, one limitation of the results obtained with splice-blocking GATA4/5 MOs is that it is unclear whether they create an equivalent of a null mutation. Nevertheless, our results show that *Xenopus *embryos have a greater and earlier requirement for GATA5 than for GATA4. A recent study by Peterkin et al, utilising translation-blocking MOs in *Xenopus laevis *has reported that GATA4 is required for cardiac morphogenesis, but not for cardiac gene expression [34]. Whilst their conclusions on the role of GATA4 in morphogenesis are in broad agreement with our study, our work suggests that GATA4 MOs also affect cardiac gene expression. It is possible that this apparent discrepancy reflects the unknown effect of translation-blocking MOs on endogenous GATA4 protein. As in the case of GATA5 stated above, the precise role of GATA4 in early development of *Xenopus *and zebrafish will be better defined when embryos lacking GATA4 become available.

The role of GATA4 in heart and liver development in *Xenopus *embryos revealed by our study appears to be in good agreement with the results from zebrafish and mouse embryos [9,11,12,26,34]. GATA4 MOs frequently result in embryos with cardia bifida (Fig. 4), a defect that also characterises GATA4 null mouse embryos [5,7]. In the mouse model, the cardia bifida phenotype is caused by GATA4-defective extraembryonic endoderm [6]. It is of note that in our *Xenopus *study specific downregulation of GATA4 in anterior endoderm also affects heart morphogenesis (Fig. 6). It is possible that these distinct forms of endoderm may share signalling activities despite being of different embryological origin.

An additional conserved feature of the action of GATA4 in development of heart and liver in *Xenopus *is its dose-dependent interaction with GATA6, which has been shown by genetic analyses of compound heterozygous mice and by MOs in zebrafish [12,38]. It is intriguing to speculate that the mechanism of this genetic interaction involves direct interaction and synergism of GATA4 and GATA6 proteins, as suggested by in vitro studies [39].

### Development of heart and liver and GATA factors

In our study we have assessed the roles of GATA4 and GATA5 in early heart and liver development. Remarkably, the vast majority of morphants for either GATA factor displayed reduced or altered expression of both heart and liver markers. One implication of these results is that the development of heart and liver are closely linked. In amniotes it has been shown that cardiac mesoderm induces liver fate in adjacent foregut endoderm [40,41], and conversely, endoderm is required for cardiac morphogenesis, as revealed by GATA4 mutant mice [5-7]. Finally, work from *Xenopus *and chick models have established a role for anterior endoderm, which is fated to become liver, in cardiac specification [42-44]. The fates of heart and liver appear to be closely intertwined through reciprocal interactions and GATA factors may mediate at least some of those. Our mosaic analyses of GATA4 and GATA5 function suggests that they can act in anterior endoderm to affect heart morphogenesis non-cell autonomously. These results are in good agreement with the null phenotype of GATA4 mice, which display heart defects caused by deficiency of GATA4 in endoderm [6]. More recent studies of the function of GATA4 in the myocardium of the mouse embryo have also demonstrated a later cell-autonomous role in growth of the heart [9], and we anticipate a similar role for *Xenopus *GATA4. However, given that the resolution of our experiments has been limited by the unknown efficacy of MOs targeted to a small part of the embryo, and by difficulty in uniquely targeting cardiac precursors by injections, better understanding of cell-specific role of GATA factors in *Xenopus *will require genetic and transgenic approaches [45,46].

### Splice-site blocking morpholino oligonucleotides as a tool for *in vivo* structure-function mapping

In this report we have shown that splice-site blocking MOs can be used to study the role of C-terminal Zn finger domain of GATA4 and GATA5 in development. This was possible because functional domains of GATA4 and GATA5 are encoded by distinct exons, and because for both genes exons 3 and 5 are in the same coding frame. Our results suggest that the C-terminal Zn finger DNA binding domain is essential for the function of GATA4 and GATA5 in heart and liver development. However, as the C-terminal Zn finger of GATA4 also determines interactions with MADS box and Nkx2.5 binding partners [47], the relative contribution of DNA- and protein-interactions to the function of this domain in embryos is not clear at present.

## Conclusion

The main finding of this work is that in *Xenopus *embryos GATA5 plays an early, essential and dose-dependent role in the development of the heart and liver. Downregulation of GATA5 causes severe reduction in the number of heart and liver precursors at the time of or shortly after their specification. In addition, we have found that in *Xenopus *GATA4 is required for heart and liver development only later after their specification. These results have confirmed and significantly extended previous studies on the roles of GATA5 and GATA4 in early vertebrate development.

## Methods

### *Xenopus* embryos and injections

*Xenopus laevis *embryos were obtained, cultured and injected with capped mRNA, DNA, MOs and a mix of rhodamine dextran and biotinylated dextran (Invitrogen UK) lineage tracer as described [36,48,49]. MOs were synthesized by Gene Tools, LLC (Oregon, USA). They were resuspended in 5 mM HEPES, pH 7.6 followed by gel filtration through Sephadex G-25 column (GE, UK). The sequences of all new MOs used are given in Additional File 1. GATA6 MO was previously described [14]. Injections of G4/5 MOs have the same effect on heart and liver development when injected either uniformly or in the anterior part of the embryo at the 4- or 8-cell stage. *X. tropicalis *embryos were obtained and injected according to  and [50]. Treatment with cycloheximide (10 μg/ml) and dexamethasone (2 μM; both from Sigma UK) in Fig. 4D was for 3 hours at 21°C.

Cell autonomy of GATA4 and GATA5 was examined by injecting 2 nl of effective concentration of SP MOs (0.9 mg/ml for G5SP and 8 mg/ml for G4SP) in anterior-vegetal blastomeres of 32/64 cell stage cardiac actin/GFP transgenic embryos [33] together with rhodamine dextran lineage tracer. Embryos were viewed and imaged on Leica MZ16F microscope with Leica DFC300 FX camera and images were processed with Adobe Photoshop 6.

### Luciferase reporter assays

1 ng of mRNA was injected with 30 pg each of 2XGATA-Luc [51] and TK-RL (Promega) and Dual Luciferase Assays (Promega UK) were carried out on extracts from 20 animal caps per sample that were incubated for 3 hours after excision at st. 9.

### Gene expression analyses

Whole mount in situ hybridisation (WMISH) was performed as described [48] with the following probes: for myocardium MLC2 [20] and cardiac Troponin I [21]; for tadpole liver primordia FOR1 [22] and Hex [23], for neurula/tailbud stage liver precursors Hex and for heart field Nkx2.5 [28]. BCIP and BM purple alkaline phosphatase substrates (Roche) were used. After bleaching [48] injected biotinylated dextran was revealed using ExtrAvidin-AP and Fast Red substrate (Sigma UK). To avoid under-representation of gene expression level that can occur for the second probe in double WMISH, both heart->liver and liver->heart probe order of colour development in WMISH was used, together with a swap of more and less sensitive AP substrates.

RT-PCR. RNA was isolated [52] and cDNA was synthesized with MMLV-RT according to manufacturer's recommendations (Promega UK). Primers for detection of the splicing events caused by G5SP and G4SP MOs: G5forward 5'-CTACCCCTCTGTGGAGACGA, G5reverse 5'-TCGAGCCTGTGGAAGTCTTT, G4forward 5'-ATGTCAACCCCACTTTGGAG and G4reverse 5'-GAGCTGGTG GAAGGAGTGAG. Hexprimers: forward 5'-CTGCCATCTCCCAACTCACT and reverse 5'-CCTCTTCTGTTCTGGAACCAGT. ODC (Orhithine Decarboxylase) primers: forward 5'-AACAAGCAGGCTGCTTCTGG and reverse 5'-GGCTGGGTTTATCACAGATG. The PCR products were sized in comparison with a DNA marker (1 kb+, Invitrogen UK). The identity of wt (3–4–5) and spliced (3–5) GATA4 and GATA5 cDNAs were confirmed by sequencing, performed by the Molecular Biology Unit, Cardiff School of Biosciences.

### Cloning of dGATA5

dGATA5a was cloned from Genbank L13701 DNA template [53] by PCR overlap method using Vent polymerase (NEB UK). In brief, the PCR product obtained using START (5'-GACAGATCTGGTTTGTAGCACCGGATCATGT) and reverse (5'-ACGAGGCACCAGTCTTTTCTGTGGCTTGAT) primers was combined with the product from the reaction using forward (5'-AGACTGGTGCCTCGTCCTCTGGCCA) and STOP (5'-GCACTCGAGTTAGGCAAGTGCCAGCGCGCACCA) primers. The resulting dGATA5a coding region was cloned into pCS2 vector and the sequence of the construct verified by DNA sequencing.

### Western blotting

20 animal caps per sample were collected at st. 10 and lysed in extraction buffer [54]. SDS-PAGE and Western blotting were performed with rat anti-HA-HRP monoclonal antibody (Roche UK) at 1:2000 dilution and following stripping, anti-Erk or -HSP70 antibodies (Sigma UK) were used to provide loading controls. Chemiluminescent signal detection and stripping were performed according to manufacturer's instructions (Perbio UK).

## Authors' contributions

KEH, SK and BVL carried out the experiments, TJM participated in the design of the study, BVL conceived of the study, and KEH and BVL wrote the paper. All authors have approved the final version of the paper.

## Supplementary Material

Additional file 1 Sequences of morpholinos used in this study and BLAST alignments to their targets. Click here for file

Additional file 2 **G5 MO reduces Tbx5 expression**. Injection of 5 ng of G5 MO leads to a reduction in Tbx5 expression at st. 23/24 (arrowhead), whereas 50 ng of C1 MO has no effect. Ventral views are shown. Click here for file

Additional file 3 **St. 22 Hex-expressing anterior ventral endoderm is fated to give rise to liver precursors**. **A: **st. 22 embryo, hybridised with Hex probe, is aligned along a graticule. **B: **strategy used for fate mapping the st. 22–24 Hex expressing ventral endoderm domain. In brief, embryos were aligned along the graticule using the pharyngeal arches, cement gland and a ventral protrusion as landmarks. The embryo was bisected caudal to the pharyngeal arches and the ventral protrusion and a small spot of DiI (Sigma) injected onto the exposed ventral endoderm. Bisected embryos were then grown until they reached st. 35. *In situ *hybridisation was performed to ascertain whether the position of the DiI corresponded to the position of Hexexpression. C, D: visible and fluorescent light views of st.35 half-embryos. **E: **merged images C, D. **F: ***in situ *hybridisation of the DiI labelled embryo using Hex antisense probe. Identical results were found in all samples examined (n = 5). Click here for file

Additional file 4 **High doses of GATA5 MOs reduce gastrula-stage Hex expression**. G5 and G5SP MO cause downregulation of Hex expression in gastrulae (st. 11) only at high doses (20 ng for G5 (30/37 embryos) and 44 ng for G5SP (27/33 embryos), whereas 50 ng of C1 MO has no effect (15/17 embryos with normal expression). G5 MOs cause gastrulation defects which cause a delay in blastpore closure and the shape of Hex domain of expression. Blastopore is highlighted by dashed line. Click here for file

Additional file 5 **G5SP MO causes splicing out of exon 4 until at least st. 33**. Embryos were injected with 9 ng of G5SP MO or 50 ng of C1 MO and mRNA extracted from st 25 or st 33 embryos was analysed for GATA5 splicing and ODC by RT-PCR. 3–4–5, cDNA that contains exon 4 and regions of exons 2 and 4; 3–5, cDNA without exon 4. E, control uninjected embryos. The 3–5 cDNA was only detected in embryos injected with G5SP MO. Click here for file
