# Supplementary material for: GATA4 and GATA5 are essential for heart and liver development in Xenopus embryos
Source: BMC Dev Biol. 2008 Jul 28;8:74. doi: 10.1186/1471-213X-8-74 (PMC2526999; doi:10.1186/1471-213X-8-74)
Supplement: Additional file 1 — Sequences of morpholinos used in this study and BLAST alignments to their targets. [file 1471-213X-8-74-S1.doc]

G5MO: 5’ CCGGCTGGGCATGGTTGGCAGTGAG

G5MO 1 CCGGCTGGGCATGGTTGGCAGTGAG 25

|||||||||||||||||||||||||

GATA5a 107 CCGGCTGGGCATGGTTGGCAGTGAG 83 atg: 65

G5MO 1 CCGGCTGGGCATGGTTGGCAGTGAG 25

|||||||||||||||||||||||||

GATA5b 284 CCGGCTGGGCATGGTTGGCAGTGAG 260 atg: 242

G5MO 1 CCGGCTGGGCATGGTTGGCAGTGAG 25

|||||||||||||||||||||||||

XtGATA5 108 CCGGCTGGGCATGGTTGGCAGTGAG 84 atg: 66

Xl GATA5a L13701; Xl GATA5b L13702; Xt GATA5 BC088567

G5UTR MO: 5’ GCTACAAACCTCACAGCTCCGGCTG

G5UTR 1 GCTACAAACCTCACAGCTCCGGCTG 25

|||||||||||||||||||||||||

GATA5a 56 GCTACAAACCTCACAGCTCCGGCTG 32 atg: 65

G5UTR 1 GCTACAAACCTCACAGCTCCGGCTG 25

|||||||||||||||||||| ||||

GATA5b 233 GCTACAAACCTCACAGCTCCTGCTG 209 atg: 242

(4 mismatches with XtGata5)

G4MO: 5’ CCTCATAGCCAGAGGGACCATG

G4MO 1 CCTCATAGCCAGAGGGACCATG 22

||||||||||||||||||||||

GATA4a 120 CCTCATAGCCAGAGGGACCATG 99 atg: 69

G4MO 1 CCTCATAGCCAGAGGGACCATG 22

|||||||||||| |||||||||

GATA4b 338 CCTCATAGCCAGGGGGACCATG 317 atg: 287

G4MO 1 CCTCATAGCCAGAGGGACCATG 22

|||||||| ||| ||| |||||

XtGATA4 284 CCTCATAGGCAGGGGGGCCTAG 262 atg: 233

Xl GATA4a U45453; Xl GATA4b BC071122; Xt GATA4 NM_001016949

C1 MO: 5’ GTAACGATTTGAGTTTGGTGTTCAT

C2 MO: 5’-ACCATCAATACATTCAGCACTCTCA.

G5SP MO: 5’ GAGGAGGA***CTGTGGGACGGAGAGAC (intron)***

G4SP MO: 5’ ATGCAGA***CTAAAGAGACAAGATATG (intron)***
