# Supplementary figures and images for: GATA4 and GATA5 are essential for heart and liver development in Xenopus embryos
Source: BMC Dev Biol. 2008 Jul 28;8:74. doi: 10.1186/1471-213X-8-74 (PMC2526999; doi:10.1186/1471-213X-8-74)

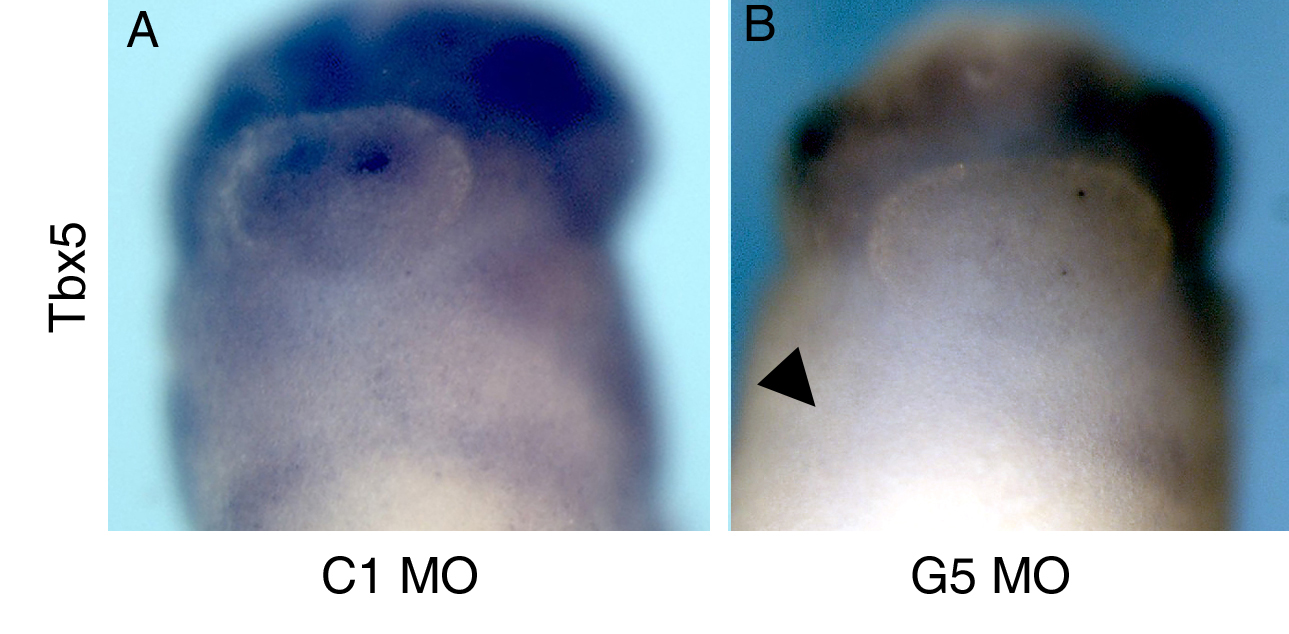

Supplement: Additional file 2 — G5 MO reduces Tbx5 expression. Injection of 5 ng of G5 MO leads to a reduction in Tbx5 expression at st. 23/24 (arrowhead), whereas 50 ng of C1 MO has no effect. Ventral views are shown. [file 1471-213X-8-74-S2.jpeg]

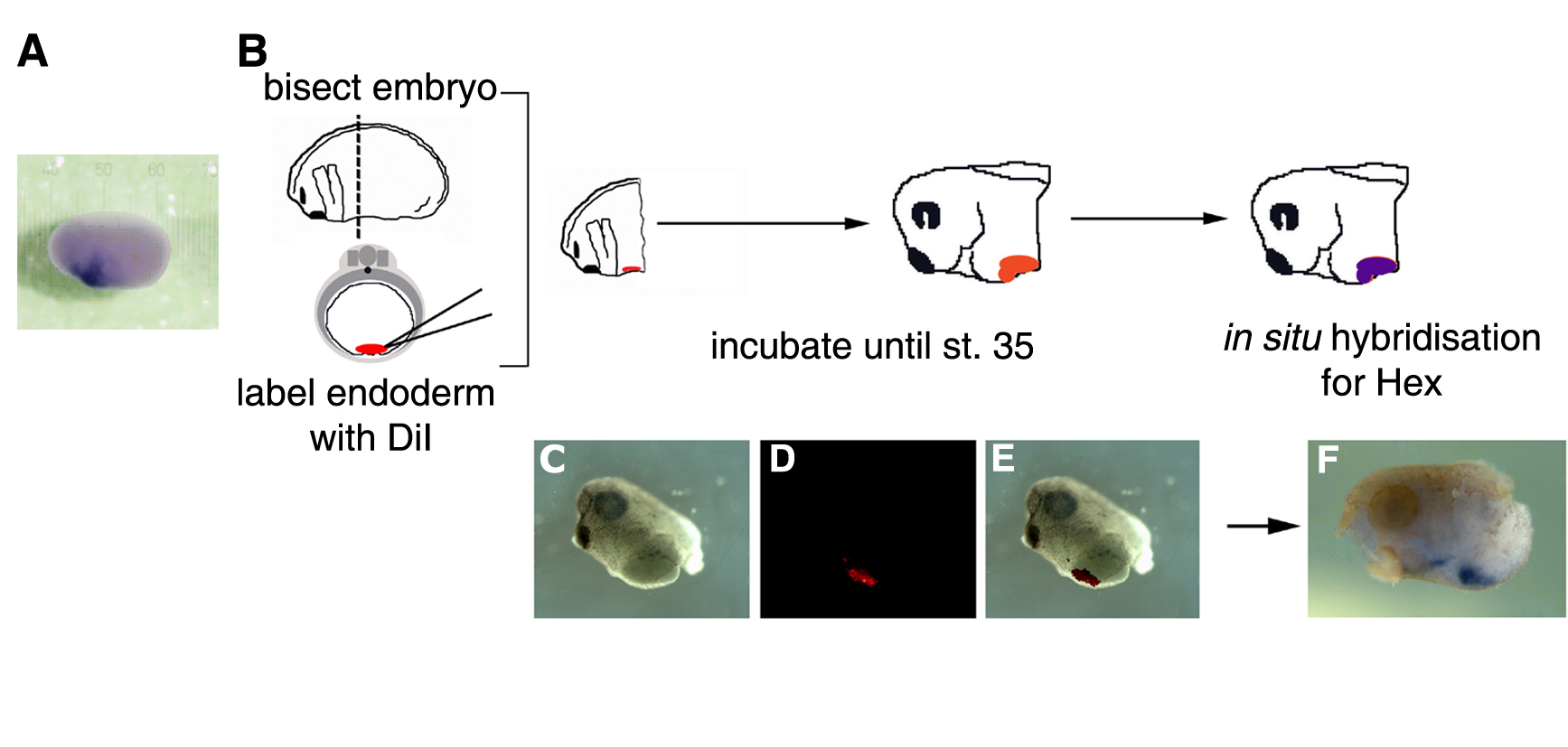

Supplement: Additional file 3 — St. 22 Hex-expressing anterior ventral endoderm is fated to give rise to liver precursors. A: st. 22 embryo, hybridised with Hex probe, is aligned along a graticule. B: strategy used for fate mapping the st. 22–24 Hex expressing ventral endoderm domain. In brief, embryos were aligned along the graticule using the pharyngeal arches, cement gland and a ventral protrusion as landmarks. The embryo was bisected caudal to the pharyngeal arches and the ventral protrusion and a small spot of DiI (Sigma) injected onto the exposed ventral endoderm. Bisected embryos were then grown until they reached st. 35. In situ hybridisation was performed to ascertain whether the position of the DiI corresponded to the position of Hexexpression. C, D: visible and fluorescent light views of st.35 half-embryos. E: merged images C, D. F: in situ hybridisation of the DiI labelled embryo using Hex antisense probe. Identical results were found in all samples examined (n = 5). [file 1471-213X-8-74-S3.jpeg]

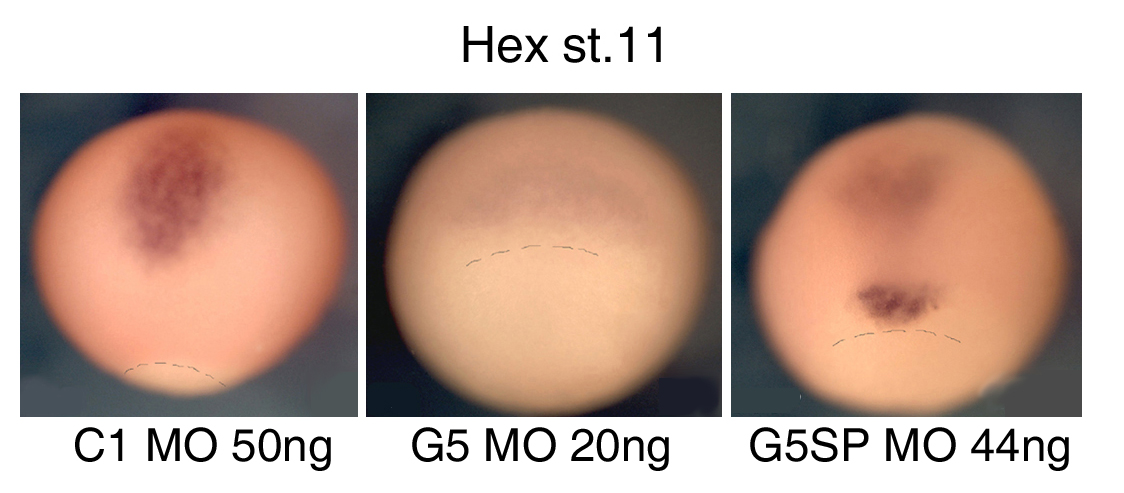

Supplement: Additional file 4 — High doses of GATA5 MOs reduce gastrula-stage Hex expression. G5 and G5SP MO cause downregulation of Hex expression in gastrulae (st. 11) only at high doses (20 ng for G5 (30/37 embryos) and 44 ng for G5SP (27/33 embryos), whereas 50 ng of C1 MO has no effect (15/17 embryos with normal expression). G5 MOs cause gastrulation defects which cause a delay in blastpore closure and the shape of Hex domain of expression. Blastopore is highlighted by dashed line. [file 1471-213X-8-74-S4.jpeg]

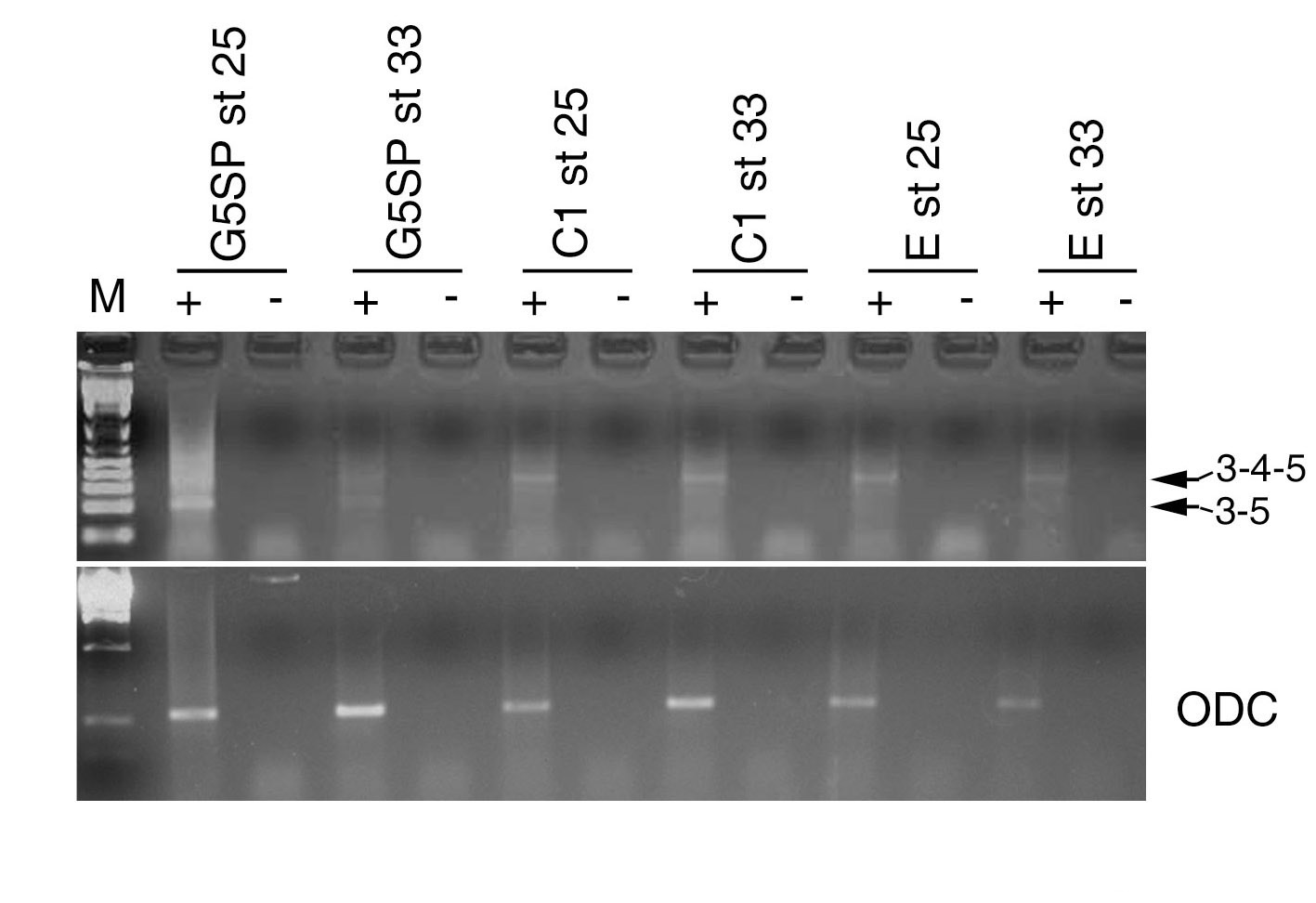

Supplement: Additional file 5 — G5SP MO causes splicing out of exon 4 until at least st. 33. Embryos were injected with 9 ng of G5SP MO or 50 ng of C1 MO and mRNA extracted from st 25 or st 33 embryos was analysed for GATA5 splicing and ODC by RT-PCR. 3–4–5, cDNA that contains exon 4 and regions of exons 2 and 4; 3–5, cDNA without exon 4. E, control uninjected embryos. The 3–5 cDNA was only detected in embryos injected with G5SP MO. [file 1471-213X-8-74-S5.jpeg]
